# Prognosis and prognostic factors of patients with mesothelioma: a population-based study

**DOI:** 10.1038/bjc.2012.245

**Published:** 2012-05-29

**Authors:** S van der Bij, H Koffijberg, J A Burgers, P Baas, M J van de Vijver, B A J M de Mol, K G M Moons

**Affiliations:** 1 Julius Center for Health Sciences and Primary Care, University Medical Center Utrecht, Heidelberglaan 100, 3584 CX, P.O. Box 85500, GA Utrecht 3508, The Netherlands; 2 Department of Thoracic Oncology, The Netherlands Cancer Institute - Antoni van Leeuwenhoek Hospital, Plesmanlaan 121 1066 CX, Amsterdam, The Netherlands; 3 Department of Pathology, Academic Medical Centre, Meibergdreef 9, Amsterdam 1105 AZ, The Netherlands; 4 Department of Cardiothoracic Surgery, Academic Medical Centre, Meibergdreef 9, Amsterdam 1105 AZ, The Netherlands; 5 Department of Biomedical Engineering, Eindhoven University of Technology, Den Dolech 2, Eindhoven 5600 MB, The Netherlands

**Keywords:** mesothelioma, survival, prognostic factors, population-based study

## Abstract

**Background:**

It is important to regularly update survival estimates of patients with malignant mesothelioma as prognosis may vary according to epidemiologic factors and diagnostic and therapeutic management.

**Methods:**

We assessed overall (baseline) survival as well as related prognostic variables in a large cohort of 1353 patients with a confirmed diagnosis of malignant mesothelioma between 2005 and 2008.

**Results:**

About 50% of the patients were 70 years or older at diagnosis and the median latency time since start of asbestos exposure was 49 years. One year after diagnosis, 47% of the patients were alive, 20% after 2 years and 15% after 3 years. Prognostic variables independently associated with worse survival were: older age (HR=1.04 per year 95% CI (1.03–1.06)), sarcomatoid subtype (HR=2.45 95% CI (2.06–2.90)) and non-pleural localisation (HR=1.67 95% CI (1.26–2.22)).

**Conclusion:**

Survival of patients with malignant mesothelioma is still limited and depends highly on patient age, mesothelioma subtype and localisation. In addition, a substantial part of the patients had a long latency time between asbestos exposure and diagnosis.

The prognosis of patients with malignant mesothelioma is usually poor. However, a small fraction of patients is still alive 2 years after diagnosis. Differences in survival are associated with age at diagnosis, gender, health status, and tumour- and environment-related factors (Burgers and Damhuis, 2004).

Several studies have shown that asbestos exposure is negatively correlated with prognosis of patients with malignant mesothelioma, and therefore prognosis can vary across regions with different histories of industrial exposure to asbestos (Spirtas *et al*, 1988; Christensen *et al*, 2008). However, geographical differences might also reflect local approaches to diagnostic and therapeutic management. Any delay in making the diagnosis of malignant mesothelioma may have a major effect on survival estimates, when survival is as short as in mesothelioma patients and can affect associations between prognostic factors and survival. Differences in prognosis resulting from differences in asbestos exposure and management of malignant mesothelioma might be expected across countries and over time. Therefore, it is important to regularly, and regionally, update survival estimates, with the use of population-based studies.

The aim of this study, based on recent evidence from a large population-based cohort in the Netherlands, was threefold: (1) to update survival estimates of patients with malignant mesothelioma, (2) to identify general predictors of survival and (3) to assess the predictive accuracy of the combined prognostic factors for prolonged survival.

## Patients and methods

### Patients

This study involves retrospective analyses of an existing registry comprising 1353 patients with malignant mesothelioma who applied to the Dutch institute for asbestos victims and entered the process for getting financial compensation between 2005 and 2008 (Baas *et al*, 2006).

After application, the standard procedure is that the patients or relatives were visited at home by a qualified representative of the Dutch asbestos institute who further explained the application procedure and compensation scheme. Patients who decided to participate in this compensation scheme had to give written informed consent for the use of their clinical data and data regarding social status, occupational circumstances and income by the institute for assessing their case and for internal and external analyses and reporting. For such linking and use of data, the most strict rules and potential sanctions are applicable regarding confidentiality and anonymisation. Accordingly, in the present analyses, data were also completely anonymised. The diagnosis of mesothelioma was confirmed by pathologists from the Dutch National Mesothelioma Panel (NMP). When pathological material was not available or insufficient for a confirmed diagnosis by the NMP (*N*=62), a final diagnosis was reached by three independent pulmonologists of the Mesothelioma Group of the Dutch Thoracic Society (Baas *et al*, 2006).

### Survival (outcome)

Survival was measured from the date of clinical diagnosis till death or censoring. The date of diagnosis was defined as the date on which malignant mesothelioma was diagnosed by the local hospital.

### Prognostic variables

The following prognostic variables were studied: gender, age at diagnosis, year of diagnosis, pathologic morphologic subtype (epithelial, sarcomatoid and biphasic type), tumour location (pleural, peritoneal or other), and various variables associated with asbestos exposure, that is, duration of asbestos exposure, latency time (defined as the time elapsed between first asbestos exposure and diagnosis) and direct exposure (yes, no).

### Analysis

For the first aim, assessing overall survival, Kaplan–Meier analyses were used and survival curves were plotted. To put results into perspective, survival probabilities of our cohort were compared with the overall survival in the general Dutch population after adjustment for gender and age.

For the second aim, associations between possible prognostic variables and survival were estimated using Cox proportional hazards regression. Missing values were imputed with multiple imputation, conforming with current guidelines, since missing values occurred on various predictor variables (see Supplementary Appendix 1) (Donders *et al*, 2006). Bootstrapping was used to correct for overfitting (Harrell, 2001; Royston *et al*, 2009).

Finally, for our third aim, we estimated the predictive accuracy of all prognostic variables combined using both discrimination and calibration statistics. The discrimination was tested by Harrell’s *c*-statistic for censored data, and corrected for overfitting (Harrell, 2001). The calibration was performed based on survival after 1 year. Predicted 1-year survival probabilities were calculated according to different prognostic factors.

All analyses were performed with SAS enterprise guide 4.3 (SAS Institute Inc., Cary, NC, USA) and R version 2.10.1 (The R Foundation for Statistical Computing, Vienna, Austria).

## Results

### Patients

The baseline characteristics of the 1353 included patients are described in Table 1 (left column). In our cohort, the mean age at the time of diagnosis was 69 years and the majority of patients were male (91%). In almost all patients (96%) the tumour was located in the pleura. Epithelial morphology was the most frequent mesothelioma type (78%). In 78% of the patients, a history of direct exposure to asbestos was identified. About half of the patients had an asbestos exposure duration ⩾20 years. The latency time since first exposure ranged from 19 to 78 years with a median of 49 years.

### Overall survival

**Figure 1:**
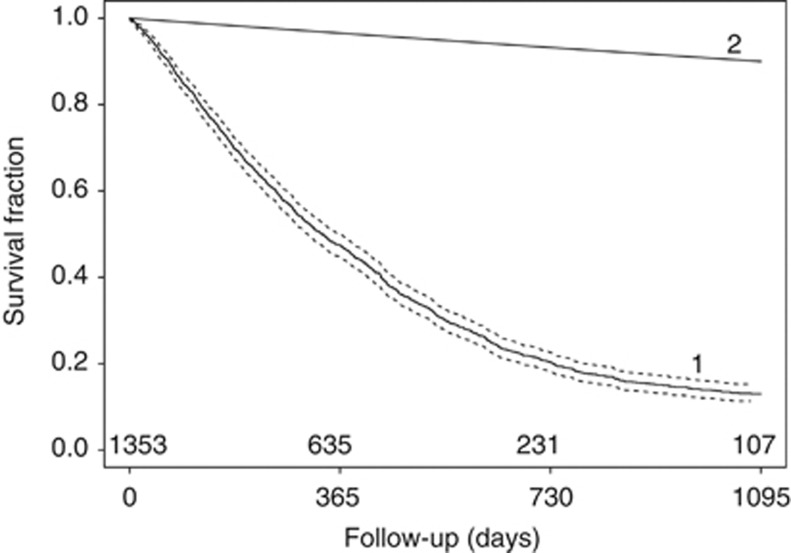
Kaplan–Meier survival curve showing the overall survival and 95% CI from the time of the diagnosis of mesothelioma for (1) the entire study cohort and (2) of the general Dutch population. The 95% CI is presented by the broken line. The Dutch population was adjusted (i.e., standardised) to the age and gender distribution of the study cohort. The number of study patients at risk is indicated at the bottom of the plot (above the *x* axis).

Figure 1 shows the overall survival curve from the time of clinical diagnosis. Median survival was 333 days (95% CI: 309–368); 47% of the patients survived longer than 1 year and 20% more than 2 years. Less than 15% of the patients were alive 3 years after diagnosis in contrast to 90% of individuals with similar age and gender distribution in the general Dutch population.

### Prognostic factors and predictive accuracy

In a univariable analysis, all variables, except direct exposure, were significantly associated with survival at a significance level of 0.05 (Table 1, middle column). In the multivariable model, only age, morphology and localisation of malignant mesothelioma had a significant independent association with survival (Table 1, last column). Hence, worse survival was independently associated with older age, sarcomatoid subtype, or non-pleural localisation.

Table 2 shows the predicted 1-year survival probabilities stratified by tumour location, pathologic subtype and age based on our multivariable model. The 1-year survival given a diagnosis of pleural malignant mesothelioma of epithelial subtype was estimated to be 77% for a patient of 50 years and 38% for a patient of 80 years. Conversely, the 1-year survival given a diagnosis of pleural malignant mesothelioma of sarcomatoid subtype was estimated to be 53% for a patient of 50 years and 9% for a patient of 80 years. These estimated survival probabilities are much lower than those in the general Dutch population, where a man aged 50 or aged 80 has a 1-year survival probability of 99.7% and 92.5%, respectively. The multivariable model showed a *c*-statistic of 0.66 (95% CI: 0.64–0.68) and very good calibration (Hosmer–Lemeshow *χ*^2^=7.63, *P*-value=0.57).

## Discussion

To date, the survival of malignant mesothelioma patients remains poor. After 1 year, only 47% of the patients were still alive. Predictors strongly associated with survival were patient age, mesothelioma localisation and subtype. These results are consistent with other population-based studies (Chapman *et al*, 2008; Mirabelli *et al*, 2009; Montanaro *et al*, 2009; Milano and Zhang, 2010). This study showed that the discriminative ability of these general predictors was moderate and the calibration was good.

Our observed survival was only marginally higher than in two older Dutch studies, in which survival among patients diagnosed with malignant mesothelioma between 1970–1994 and 1987–1989 was studied (van Gelder *et al*, 1994; Janssen-Heijnen *et al*, 1999). In these studies the probability of 1-year survival was about 42%, suggesting that survival has not improved substantially over the years. Lack of improvement was also observed by a recent Italian and American study (Montanaro *et al*, 2009; Milano and Zhang, 2010). However, if the mix of patients has changed over the years due to, for example, improved diagnosis in patients with suspected mesothelioma, then direct comparisons between older studies and our study are hard to make. Moreover, in the Dutch study of van Gelder *et al* (1994), in which patients diagnosed between 1987 and 1989 were studied, 42% of the patients were 65 years or younger, whereas in our study only 30% of the patients were younger than 65 years (data not shown).

The higher age in our cohort likely relates to currently longer latency times between asbestos exposure and diagnosis. Our results showed an average latency time of 49 years between initial asbestos exposure and diagnosis.

The prognostic value of patient age, malignant mesothelioma subtype and localisation can assist in the selection of patients more likely to benefit from intensive treatment modalities, especially for patient selection in future therapeutic randomised trials. However, in the current study not all potentially relevant predictors were available that might contribute to the discrimination of survival among malignant mesothelioma patients. For example, there is some evidence that patients’ general well-being and weight loss are important prognostic factors in patients with malignant mesothelioma (Edwards *et al*, 2000; Ak *et al*, 2009; Nowak *et al*, 2010). Therefore, we expect that predictive accuracy might improve, when these predictors would also be taken into account. We did not observe a significant association between characteristics of asbestos exposure and survival. However, in our data set asbestos exposure was mainly based on self-reporting, which could mean that exposure estimates are of lower quality than the other predictors considered.

Recently, more treatment options have become available for patients with malignant mesothelioma. Although these may benefit selected patients, their results are still far from satisfactory for the majority of the patients (Mott, 2012). In our study, patients received treatment according to latest insights, suggesting that, in general, the impact of treatment is still limited.

To improve the effect of treatment, an early diagnosis of malignant mesothelioma is of great value. This may hold in particular for patients with peritoneal mesothelioma, as the observed difference in survival between peritoneal mesothelioma and pleural mesothelioma may be explained by a delayed diagnosis of peritoneal mesothelioma due to the complexity of the disease (Manzini *et al*, 2010).

In conclusion, we showed that overall survival in patients with malignant mesothelioma remains poor and depends highly on patient age, malignant mesothelioma subtype and localisation. Additionally, we found that half of the patients are 70 years or older and a substantial part of the patients has a long latency time since asbestos exposure. A trend towards longer latency times may have profound implications for future lawsuits and reimbursements as in several countries financial compensation depends (partially) on latency times (Baas *et al*, 2006). Furthermore, the future prevalence of mesothelioma might still remain high as a result of these long latency times.

## Figures and Tables

**Table 1:** Baseline characteristics and their unadjusted and adjusted effect on survival in patients diagnosed with malignant mesothelioma

**Variables**	**Patients (** ***n*** **=1353) (values are numbers (%) unless stated otherwise)**	**Crude HR (95% CI)**	**Adjusted HR (95% CI)** a
*Age (in years)*			
<60	209 (15.4)	1.00 (Reference)	1.00 (Reference)
60–<70	499 (36.9)	1.94 (1.58–2.37)**	1.82 (1.46–2.26)**
70–<80	541 (40.0)	2.83 (2.32–3.46)**	2.47 (1.93–3.17)**
⩾80	104 (7.7)	3.83 (2.93–5.01)**	3.38 (2.45–4.65)**
Age (as continuous variable, mean±s.d.)	69 (±8)	1.05 (1.04–1.06)**	1.04 (1.03–1.06)**
Median age (min–max)	69 (39–95)		
			
*Gender*			
Female	120 (8.9)	1.00 (Reference)	1.00 (Reference)
Male	1233 (91.1)	1.40 (1.13–1.73)**	1.16 (0.93–1.46)
			
*Tumour location*			
Pleural	1296 (95.8)	1.00 (Reference)	1.00 (Reference)
Non-pleural/peritoneal^b^	57 (4.2)	1.39 (1.05–1.85)**	1.67 (1.26–2.22)**
			
*Mesothelioma morphology (pathologic subtype)*			
Epithelial	1049 (77.5)	1.00 (Reference)	1.00 (Reference)
Sarcomatoid	209 (15.4)	2.50 (2.13–2.93)**	2.45 (2.06–2.90)**
Mixed	95 (7.0)	1.59 (1.27–1.99)**	1.65 (1.32–2.06)**
			
*Asbestos exposure*			
Duration of asbestos (in years)			
<5 years	143 (10.6)	1.00 (Reference)	1.00 (Reference)
5–<10 years	164 (12.1)	1.12 (0.87–1.44)	1.18 (0.92–1.52)
10–<20 years	423 (31.3)	1.21 (0.98–1.49)	1.21 (0.98–1.49)
20–<30 years	321 (23.7)	1.25 (1.00–1.58)	1.19 (0.95–1.48)
⩾30 years	302 (22.3)	1.59 (1.27–1.98)**	1.28 (1.02–1.61)*
Duration (as continuous variable, mean±s.d.)	20 (±12)	1.01 (1.01–1.02)**	1.00 (1.00–1.01)
Median duration (min–max)	19 (1–66)		
Latency time (in years)			
<40 years	268 (19.8)	1.00 (Reference)	1.00 (Reference)
40–<50 years	487 (36.0)	1.24 (1.03–1.48)**	0.94 (0.77–1.15)
⩾50 years	598 (44.2)	1.98 (1.66–2.36)**	1.10 (0.86–1.40)
Latency (as continuous variable, mean±s.d.)	48 (±9)	1.03 (1.03–1.04)**	1.00 (0.99–1.01)
Median latency (min–max)	49 (19–78)		
Direct exposure of asbestos^c^	1052 (77.8)	1.14 (0.99–1.32)	1.01 (0.87–1.17)

Abbreviations: CI=confidence limit; HR=hazard rates.

*Significant at a *P*-value of 0.05.

**Significant at a *P*-value of 0.01 (the overall *P*-value was also checked for categorical variables with more than two categories and was significant in the multivariable model for age and pathologic subtype (*P*<0.01)).

Overall, the most significant predictor in the multivariable model was pathologic subtype.

a HRs after shrinking, results of the multivariable model are based on the inclusion of the continuous variables as linear terms, the model was refitted for the estimation of the HRs of the continuous variables as categorical variables.

b Including one patient with pericardial mesothelioma, all other patients had peritoneal mesothelioma.

c In comparison with second-hand exposure and no distinct asbestos exposure.

**Table 2:** Predicted 1-year survival from the time of the diagnosis of mesothelioma stratified by tumour location, pathologic subtype and age^a^

	**Predicted 1-year survival of patients with pleural mesothelioma (%)**	**Predicted 1-year survival of patients with peritoneal mesothelioma (%)**
*For epithelial subtype (age in years)*		
50	77	65
60	67	51
70	53	35
80	38	20
		
*For sarcomatoid subtype (age in years)*		
50	53	34
60	37	19
70	22	8
80	9	2

a Results were based on the average values over the other covariates.
