# Supplementary material for: Prognosis and prognostic factors of patients with mesothelioma: a population-based study
Source: Br J Cancer. 2012 May 29;107(1):161–4. doi: 10.1038/bjc.2012.245 (PMC3389430; doi:10.1038/bjc.2012.245)
Supplement: Supplementary Appendix 1 [file bjc2012245x1.doc]

**Appendices (online only)**

Appendix 1. Distribution of missing values among available variables (including the outcome).

| **Variables** | **Missing values**  number (%) | **Complete cases**  (patients with all variables completely observed)  n=686 (51%) | **Patients with at least one missing value**  n=667 (49%) | **P-**  **value** ^b^ |
| --- | --- | --- | --- | --- |
| **Age** *(in years) mean (± sd)* | 156 (12) | 69 (±8) | 69 (±9) | 0.294 |
| **Male gender** | 1 (0) |  |  | <.001 |
| *Female* |  | 18 (3) | 102 (15) |  |
| *Male* |  | 668 (97) | 564 (85) |  |
| **Tumour location** | 90 (7) |  |  | 0.001 |
| *Pleural* |  | 667 (97) | 539 (93) |  |
| *non-pleural/ peritoneal* |  | 19 (3) | 38 (7) |  |
| **Mesothelioma morphology *(pathologic subtype)*** | 72 (5) |  |  | 0.880 |
| *Epithelial* |  | 520 (76) | 457 (77) |  |
| *Sarcomatoid* |  | 113 (16) | 96 (16) |  |
| *Mixed* |  | 53 (8) | 42 (7) |  |
| **Asbestos exposure** |  |  |  |  |
| *mean duration of exposure*  *in years (± sd)* | 314 (23) | 20 (±13) | 20 (±14) | 0.545 |
| *mean latency time in years (± sd)* | 360 (27) | 48 (±9) | 48 (±9) | 0.830 |
| *direct exposure of asbestos* | 523 (39) | 574 (84) | 67 (47) | <.001 |
| **Survival** *mean time in days (± sd)* | 156 (12) ^a^ | 444 (±395) | 424 (±385) | 0.403 |

^a^ missings are due to missing data on the actual date of diagnosis ; ^b^ Significant (Underlined) P-values indicate that missing data were clearly not missing completely at random (MCAR) but related to the observed variables. Hence, patients with missing values were no random but rather a selective subset of the total cohort. Simply excluding this selective subset of subjects by performing a so-called ‘complete subject analysis’ would thus lead to biased results. Therefore, missing values were imputed with multiple regression techniques.
